# Novel Metformin-Based Schiff Bases: Synthesis, Characterization, and Antibacterial Evaluation

**DOI:** 10.3390/ma13030514

**Published:** 2020-01-22

**Authors:** Inas Al-Qadsy, Waseem Sharaf Saeed, Abdel-Basit Al-Odayni, Lena Ahmed Saleh Al-Faqeeh, Abdulaziz Ali Alghamdi, Mazahar Farooqui

**Affiliations:** 1 Maulana Azad of Arts, Science and Commerce, P.O. Box 27, Aurangabad 431001, India; inas2015228@gmail.com; 2 Chemistry Department, College of Science, King Saud University, P.O. Box 2455, Riyadh 11451, Saudi Arabia; wsaeed@ksu.edu.sa (W.S.S.); aalghamdia@ksu.edu.sa (A.A.A.); 3 Microbiology Department, Dr. Babasaheb Ambedkar Marathwada University, P.O. Box 27, Aurangabad 431004, India; lenaalfaqeeh8@gmail.com

**Keywords:** Schiff bases, metformin hydrochloride, green synthesis, antibacterial activity

## Abstract

Novel Schiff bases of metformin hydrochloride and (*ortho*)*para*-nitrobenzaldehyde were synthesized by employing two efficient environmentally friendly methods, namely, stirring and microwave-assisted methods using water as the solvent. The advantage of microwave irradiation over the other methods was represented in the reduction of reaction time and wastes, and good yields; however, water in both methods plays the role of eco-friendly solvent. The structural properties of the (*ortho*)*para*-isomer products were analyzed by elemental analysis, Fourier transform infrared (FTIR) spectroscopy, UV-Visible (UV-Vis) spectroscopy, ^1^H nuclear magnetic resonance (NMR) spectroscopy, ^13^C NMR spectroscopy, mass spectroscopy, and differential scanning calorimetry (DSC). The newly synthesized compounds were screened for their antibacterial activity against selected Gram-positive (ATCC 25923, ATCC 43300, and ATCC 29212) and Gram-negative (ATCC 25922, ATCC 27853, and ATCC 700603) bacteria using the agar well diffusion method. Compared with the standard drug streptomycin, both Schiff bases exhibited moderate bactericidal activity against the tested bacteria with higher values of *ortho*-nitro compared with the *para*-nitro isomer; however, no effect on ATCC 43300 and ATCC 27853 was observed under the experimental conditions employed.

## 1. Introduction

Organic compounds with nitro groups are a class of bioactive drugs, which they are naturally or synthetically available. Many of these compounds have a great therapeutic significance; thus, they are important substances in pharmaceutical and medicinal applications. Compounds with nitro groups are commonly implicated in the treatment of many diseases, including cardiovascular diseases, Parkinson’s disease, peptic ulcers, and cancer. Such compounds also possess antimicrobial potency, including antibacterial effect against some pathogenic Gram-positive and Gram-negative bacteria such as Salmonella, Klebsiella, Escherichia, Enterobacter, Vibrio genus, and Shigella [1].

Metformin hydrochloride is an N, N-dimethyl biguanide glucose-lowering agent that was extracted from the plant Galega officinalis in the 1920s; further, metformin (Mf) has been widely used to control noninsulin-dependent diabetes mellitus (NIDDM) [2,3,4]. Diabetes mellitus (DM), generally called diabetes, is a group of metabolic disorders characterized by high blood sugar levels over a long time; this results in symptoms such as increased thirst and hunger as well as frequent urination. Metformin improves liver sensitivity to insulin, decreases glucose production in the liver, increases the absorption of insulin, and induces the consumption of glucose by peripheral tissues; therefore, it is effective in treating the loss of appetite, which leads to a decrease in weight. Besides its utilization as an antidiabetic, metformin has been shown to have anti-cancer and anti-aging effects and a risk factor reducer for cardiovascular disease [5,6,7,8]. Although other biguanide drugs induce lactic acidosis, metformin does not; however, nearly 30% of patients on metformin therapy suffer from gastrointestinal side effects [9].

Metformin is ingested by mouth; this is done usually 2–3 times per day (high dose) to enable glucose-lowering. It is a strong base with pKa = 11.5; therefore, ionized metformin has a high polarity and a tendency to adsorb negatively charged intestinal epithelia. This absorption decreases drug absorption, which causes fast renal elimination and poor significant metabolism. However, the pharmacodynamics shows no relationship between plasma drug concentration and the magnitude of effect [10,11,12]. Additionally, several unexpected advantages have been detected for metformin as an anti-cancer drug. It has been discovered through epidemiological studies that there is a relationship between type Ⅱ diabetes (NIDDM), human insulin- or insulin analog-based diabetes treatment, and the development of some cancers such as breast, pancreas, colon, and rectum, as well as kidney [13,14,15,16,17,18,19,20].

It has recently been found that metformin can be used in the treatment of cancer cells similar to known cancer drugs such as doxorubicin, cisplatin, and paclitaxel. Moreover, the interaction of metformin with other anti-cancer drugs improves their efficacy and reduces the toxicity in cells [21,22,23,24,25,26,27]. Antibacterial activity of the free metformin and some of its derivatives, including Schiff bases and complexes with some metals, has been evaluated against selected bacteria strains such as *Streptococcus, Staphylococcus, Proteus mirabilis, Escherichia coli,* and *Bacillus megaterium* [28,29]. The results revealed no or poor activities of the metformin-based ligands compared to the metformin, while a higher activity was observed of their complexes against these bacteria.

Since there is an increase in the number of people suffering from DM in recent years, numerous researchers have focused on the synthesis of new antidiabetic drugs such as Schiff bases from metformin [11,30,31,32,33,34,35,36].

In this study, we synthesized two new Schiff bases derived from metformin hydrochloride, 2-nitrobenzaldehyde, and 4-nitrobenzaldehyde via an eco-friendly methodology. Compounds 1 and 2 were successfully synthesized and characterized using elemental analysis, Fourier transform infrared (FTIR) spectroscopy, UV-Visible (UV-Vis) spectroscopy, ^1^H nuclear magnetic resonance (NMR) spectroscopy, ^13^C NMR spectroscopy, mass spectroscopy, and differential scanning calorimetry (DSC). The antibacterial activity of both metformin-derivative Schiff bases was evaluated against a range of Gram-positive and Gram-negative bacteria using an agar well diffusion method.

## 2. Materials and Methods

### 2.1. Chemistry—General

All chemicals and solvents employed in this study were commercially available and were used as received. Metformin hydrochloride (C_4_H_11_N_5_.HCl) was purchased from Sigma-Aldrich. 2-nitrobenzaldehyde (99%) was obtained from SD Fine Chem Limited. 4-nitrobenzaldehyde (99%) was purchased from Research-Lab Fine Chem Industries.

The completion of the reaction was monitored by thin-layer chromatography (TLC) using ethyl acetate and dichloromethane (8:2 volume ratio) as the mobile phase. The elemental composition was obtained using a EuroEA3000 CHNS-O analyzer (EuroVector S.p.A. (EVISA), Milan, Italy). Melting points were determined using a CL-726 digital apparatus (IndiaMART Member Since, Noida, India). The DSC analysis was performed on a Shimadzu DSC 60A system (Kyoto, Japan), which was calibrated with indium before use. A sample of 8–10 mg was deposited in an aluminum pan and closed before being placed in the DSC analysis cell. All samples were scanned from 25 to 300 °C in a nitrogen atmosphere at a heating rate of 20 °C⋅min^−1^. The melting points were measured during the second scan. FTIR analyses were carried out using a Nicolet iS10 spectrophotometer (Thermo Scientific, Waltham, MA, USA) equipped with an attenuated total reflection (ATR; diamond crystal) accessory. All scans were performed over the 4000–500 cm^−1^ range, with a total of 32 scans per spectrum and spectral resolution of 4 cm^−1^. The electronic spectra were recorded in dimethyl sulfoxide/water (2:8 by volume) using a double beam UV-Vis spectrophotometer (U-210, Hitachi, Tokyo, Japan) at room temperature over the wavelength range of 600–200 nm. ^1^H NMR and ^13^C NMR spectra were recorded at 500 MHz and 200 MHz, respectively, using a JEOL ECP400 NMR spectroscope (Tokyo, Japan). The mass spectrum was obtained using an Accu-TOF LC-plus JMS-T100 LP atmospheric pressure ionization ToF-MS spectrometer (JEOL, Tokyo, Japan) equipped with a direct analysis in real-time (DART) ion source (IonSense, Saugus, MA, USA) and operated in the positive-ion mode. Selected peaks were assigned using Mass Centre software (version 1.3.m).

### 2.2. Synthesis of Metformin Schiff Bases

#### 2.2.1. Conventional Method

A mixture of equimolar amounts (10 mmol each) of metformin-HCl and the (*ortho*)*para*-substituted benzaldehyde was prepared in 20 mL of methanolic basic media and refluxed for 2–3 h. The progress of the reaction was monitored by TLC. After reaction completion, the pale to intense yellow-colored solutions were poured on ice, filtered, dried, and recrystallized from ethanol.

#### 2.2.2. Eco-Friendly Methods

Two novel eco-friendly methods were established for the synthesis of the metformin-based Schiff bases with the (*ortho*)*para*-nitrobenzaldehyde and compared with the above-mentioned conventional method. Typically, a solution of equimolar amounts (10 mmol each) of metformin and nitro-substituted benzaldehydes was prepared in a basic aqueous media. The mixture was stirred using a magnetic stirrer at room temperature (Method I) or refluxed under microwave irradiation (Method II) until reaction completion, as indicated by TLC. Then, the products were collected and purified following the conventional method procedure.

### 2.3. Biological Tests

#### 2.3.1. Bacterial Isolates

Standard bacterial strains of Gram-positive (*Staphylococcus aureus* (ATCC 25923) (*S. aureus*), methicillin-resistant *Staphylococcus aureus* (ATCC 43300) (*MRSA*), *Enterococcus faecalis* (ATCC 29212) (*E. faecalis*)), Gram-negative (*Escherichia coli* (ATCC 25922) (*E. coli*), *Pseudomonas aeruginosa* (ATCC 27853) (*P. aeruginosa*), and *Klebsiella pneumoniae* (ATCC 700603) (*K. pneumoniae*)) bacteria were collected from the Microbiology Department, Medical College, Aurangabad, India.

#### 2.3.2. Antibacterial Activity of Metformin Schiff Bases

*In vitro* antibacterial activity was carried out using the agar well diffusion method [37,38]. Metformin Schiff bases (Comp1 and Comp2) were dissolved in dimethyl sulfoxide (DMSO) to a final concentration of 1–10 mg⋅mL^−1^; DMSO, however, has been used as a negative control as well and has shown no inhibition against the tested organism [39]. Nutrient agar plates were prepared following a typical method then allowed to solidify. First, 100 µL of bacterial suspension (adjusted to 0.5 MacFarland turbidity standard) were spread on the surfaces of the plates using cotton swabs. Wells of 8 mm in diameter were made on the agar plates using a sterile cork borer. Then, 100 µL of Comp1, Comp2, the positive control (Streptomycin), and the negative control (DMSO) were introduced into the wells and labeled. The plates were incubated at 37 °C for 18–24 h. The diameters of the inhibition zones were measured in millimeters. Studies were performed in duplicate, and the data were averaged. Statistical analysis of the data was performed by one-way ANOVA using SPSS ver. 20.0 software and the data were reported as the mean and the standard error of the mean (SEoM).

## 3. Results and Discussion

**Scheme 1:**
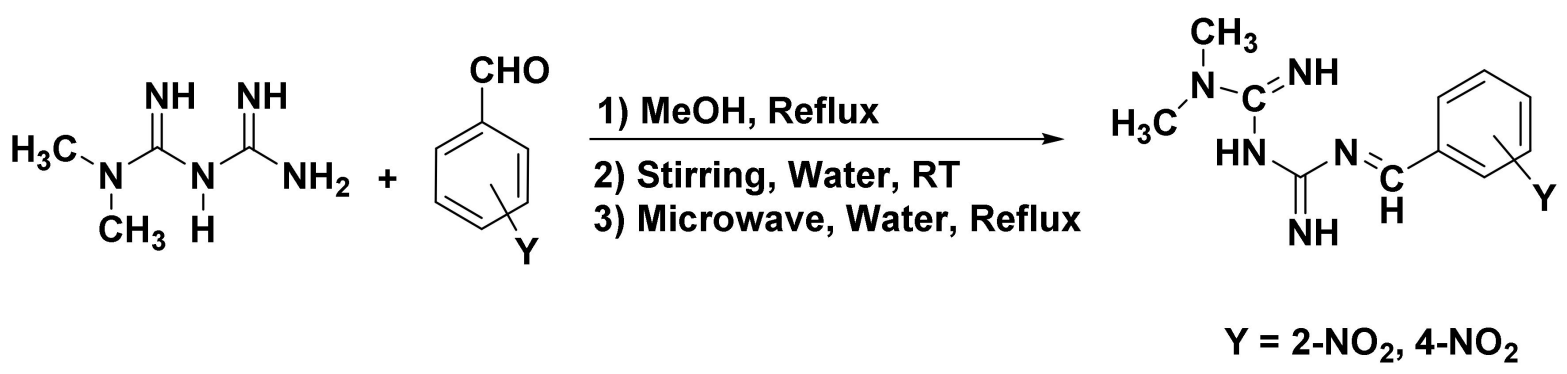
Synthesis of metformin Schiff bases.

The Schiff base formation of metformin hydrochloride with *nitro*-substituted benzaldehyde was successfully carried out using conventional, stirring, and microwave-assisted methods. The reaction route is given in Scheme 1.

As shown in Table 1, the reaction time was substantially reduced in the microwave method compared to the others. On the other hand, as the stirring method was performed at room temperature, the reaction time was longer (10 h). However, the stirring and microwave-assisted methods were carried out in an aqueous reaction medium, which is undoubtedly an eco-friendly solvent. Moreover, the yield obtained from the three methods were in the order of microwave-assisted > stirring > conventional, indicating that the new methods were convenient and could be used for the practical synthesis of metformin-based Schiff bases.

### 3.1. Structure and Spectral Analysis

**Figure 1:**
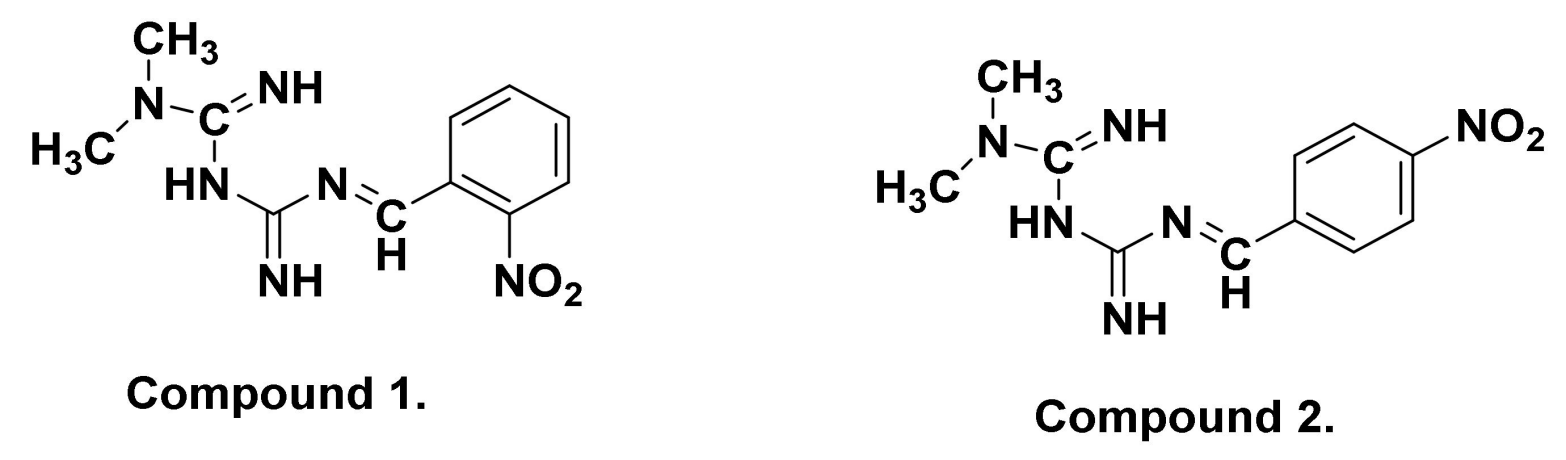
Chemical structures of metformin Schiff bases Comp1 and Comp2.

The structure of Comp1 and Comp2 (Figure 1) was confirmed by elemental analysis, FTIR, ^1^H NMR, ^13^C NMR, UV-Vis, and mass spectroscopy. In addition to the TLC technique used for reaction monitoring and purity evaluation, the purity of the compounds was also confirmed by DSC.

#### 3.1.1. Elemental Analysis

The elemental analysis of the synthesized metformin-based Schiff bases (Figure 1) was performed using a CHNSO analyzer, and the results, along with the calculated values, are given in Table 1. The results indicate perfect agreement between the experimental and calculated percentages of the C, H, N, and O atoms in both compounds, confirming their chemical structures.

#### 3.1.2. FTIR Analysis

**Figure 2:**
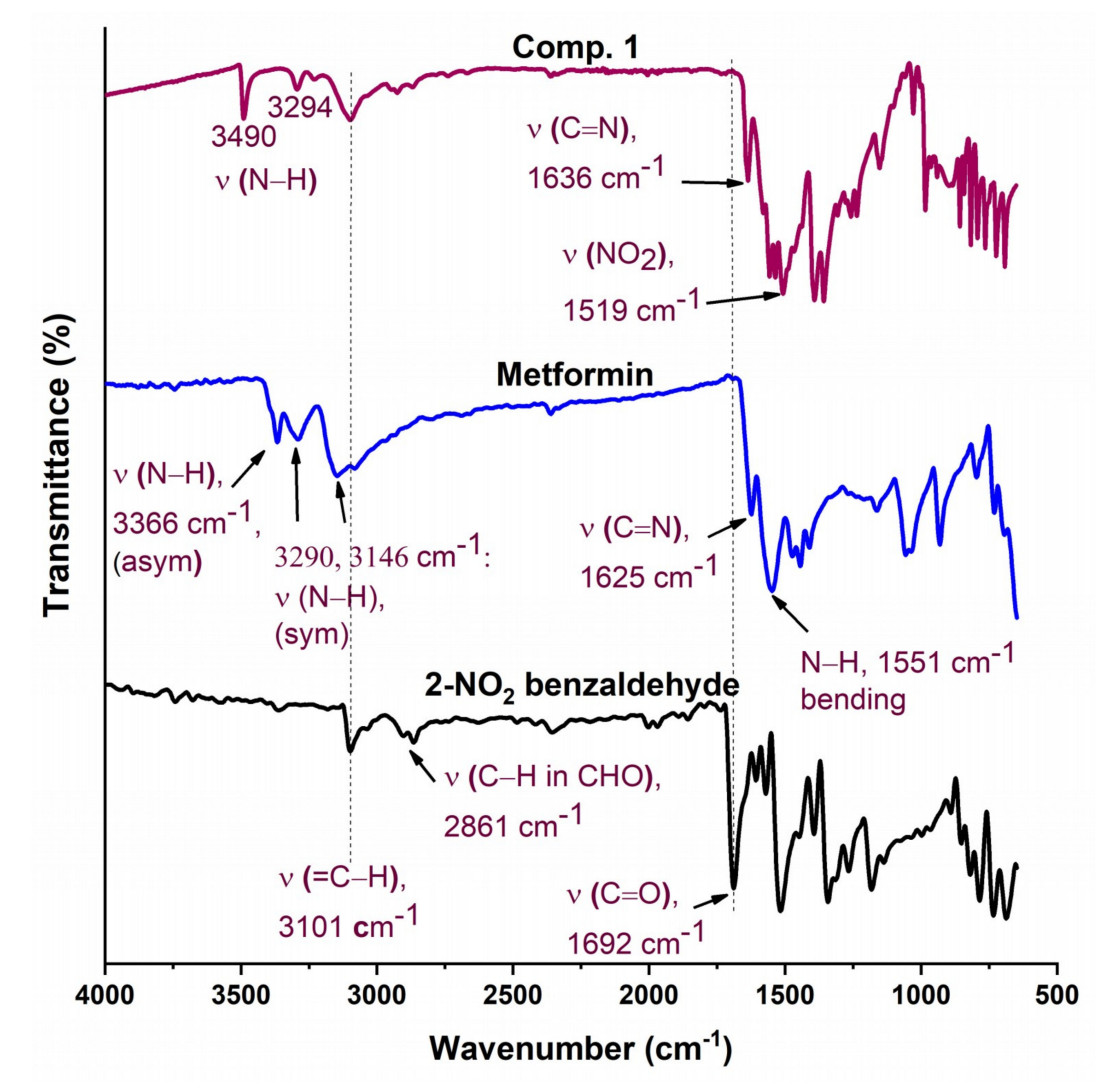
Fourier transform infrared (FTIR) spectra of Comp1, pure metformin-HCl, and *ortho*-nitrobenzaldehyde.

**Figure 3:**
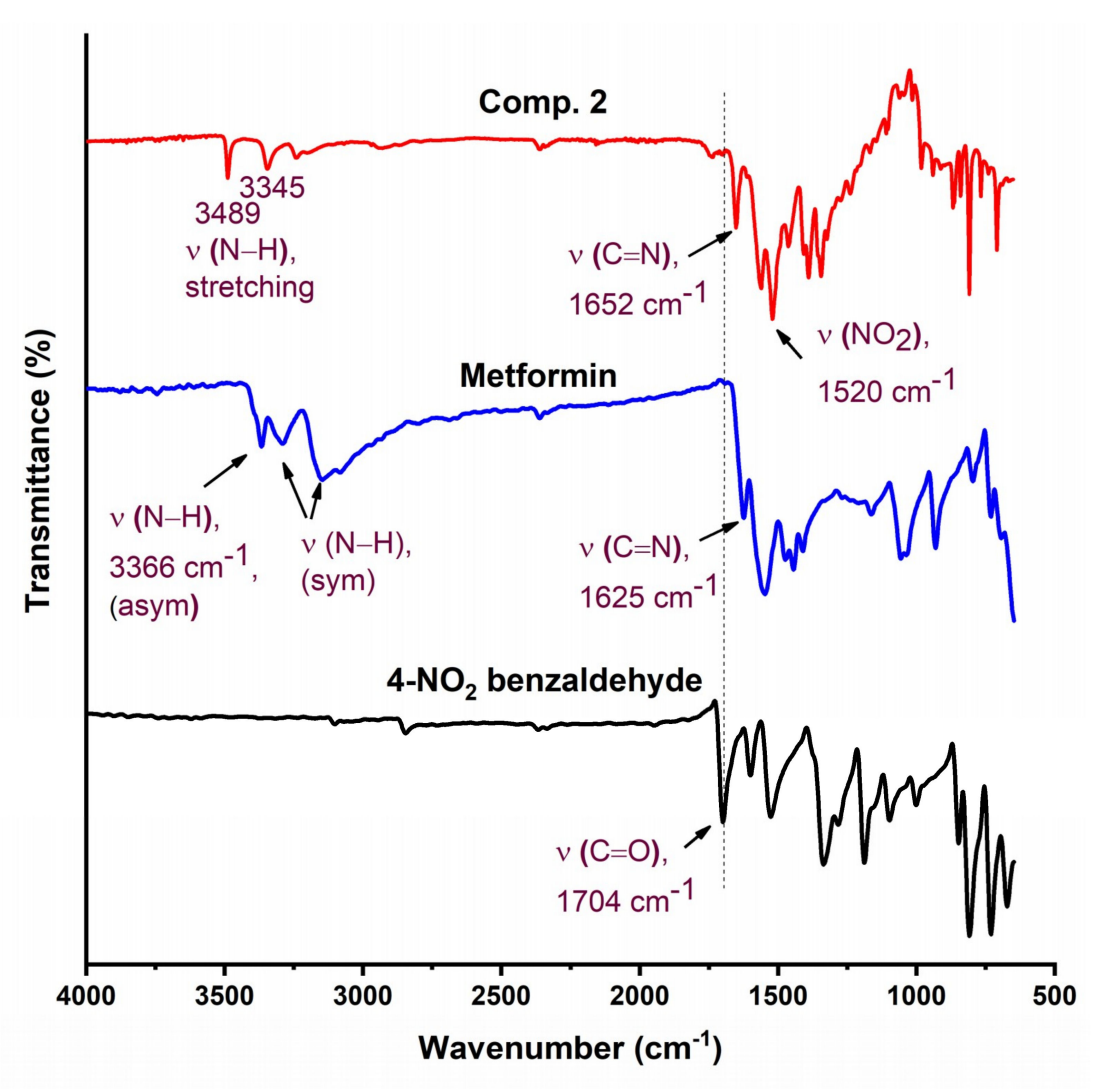
FTIR of Comp2, pure metformin HCl, and 4-nitrobenzaldehyde.

The FTIR spectra of Comp1 and Comp2 are shown in Figure 2 and Figure 3 along with their precursors for comparison. In Figure 2, the spectra of both starting materials (metformin and 2-nitrobenzaldehyde) are in good agreement with spectra reported in the literature [40,41]. Bands corresponding to asymmetric and symmetric stretching vibrational modes of primary metformin amines (NH_2_) were observed at 3366 cm^−1^ and 3290 cm^−1^, respectively. In the spectrum of Comp1, the typical pattern of the primary amine absorption peaks is not present, while that of the N–H secondary amine appears at 3490 cm^−1^, confirming the reaction success. In addition, the stretching vibration of the benzaldehyde carbonyl group, C=O, at 1692 cm^−1^ completely disappeared in the spectrum of Comp1, again confirming the reaction success. The absorption peak of the imine functional group characterizing the synthesized Schiff base is observed at 1636 cm^−1^, overlapping with the C=N metformin-originating primary imines, which is evident at 1625 cm^−1^ in the metformin spectrum. Bands corresponding to C=C stretching, C–H deformation, and NO_2_ in the Comp1 spectra are assigned between 1580 and 1507 cm^−1^ and shift to lower values compared with the metformin and benzaldehyde spectra (Figure 2). The bands corresponding to =C–H stretching of the benzene ring in 2-nitrobenzaldehyde at 3101 cm^−1^ is slightly shifted to 3099 cm^−1^ in the spectrum of Comp1. The change in the vibrational frequencies of certain bonds after Schiff base formation indicates special material characteristics such as resonance, bond environment, and hydrogen bonding.

The FTIR spectra of Comp2 is shown in Figure 3. The characteristic aldehyde band for the C=O group in 4-nitrobenzaldehyde is observed at a higher frequency (1704 cm^−1^) compared with 2-nitrobenzaldehyde (1692 cm^−1^), reflecting the bond strength and thus stability of 4-nitrobenzaldehyde over 2-nitrobenzaldehyde. Similarly, bands for C=N in a Schiff base (1652 cm^−1^) and NO_2_ (1520 cm^−1^) for Comp2 appear at higher energies compared with Comp1 (1636 and 1519 cm^−1^, respectively). However, bands corresponding to N–H bonds (the N–H of the secondary amine and primary imine) are observed at lower frequencies in Comp2 compared with Comp1. This result is due to the structural differences between the *ortho*- and *para*-substituted aromatic rings. The structure of Comp2 can be followed by the disappearance of the NH_2_ and C=O absorption bands in the Comp2 spectra compared with metformin and benzaldehyde, respectively, and subsequent appearance of the C=N azomethine (Schiff base) peak at 1652 cm^−1^ [42,43,44,45].

#### 3.1.3. ^1^H NMR Analysis

**Figure 4:**
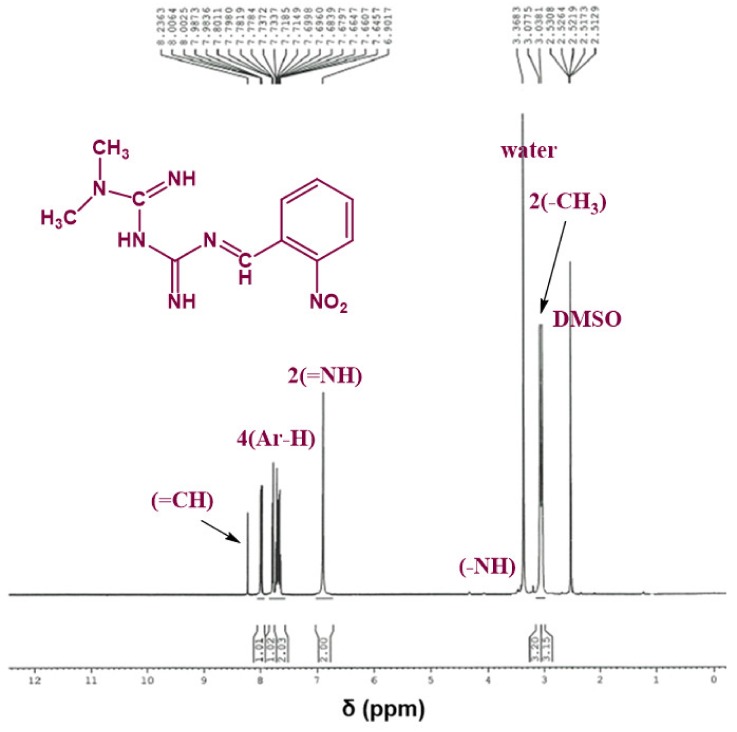
^1^H NMR spectrum of Comp1 in DMSO-*d*6.

**Figure 5:**
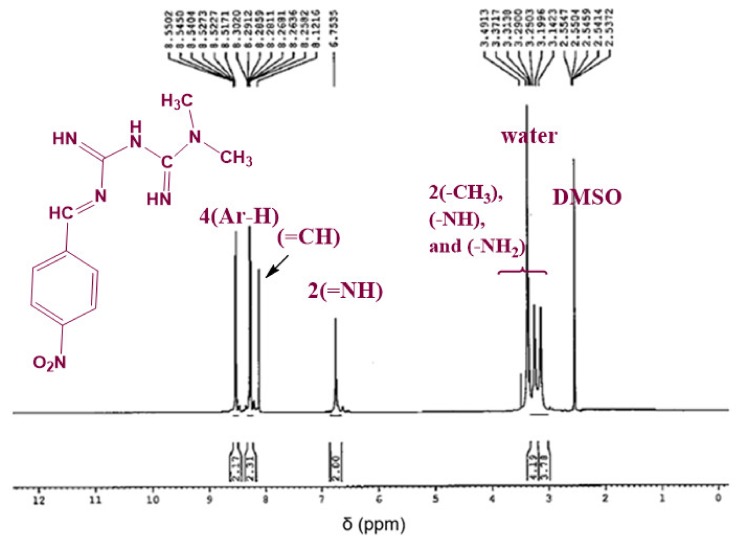
^1^H NMR spectrum of Comp2 in DMSO-*d*6.

The chemical structure of the synthesized Schiff bases was also confirmed via NMR spectroscopy. Figure 4 shows the ^1^H NMR spectrum of Comp1 in which the integrated peaks reflect the number of protons in the molecule. The two methyl groups are observed as singlet peaks at 3.038 and 3.078 ppm, while the proton corresponding to the new imine appears as a singlet at 8.236 ppm. The characteristic bands of the aromatic protons (Ar–H) are seen in the range of 8.006–7.646 ppm. The metformin-based protons of primary imines (=NH) are observed at 6.902 ppm. In addition, the singlet peak at 3.368 ppm is assigned to the secondary amine proton. Similarly, the protons corresponding to Comp2 are assigned in Figure 5. However, the extra peaks observed in the range of 3.142–3.491 ppm are possibly due to the overlapping of the molecular peaks with water. The position of all the other protons is evident in the respective figures [45,46].

#### 3.1.4. ^13^C NMR Analysis

**Figure 6:**
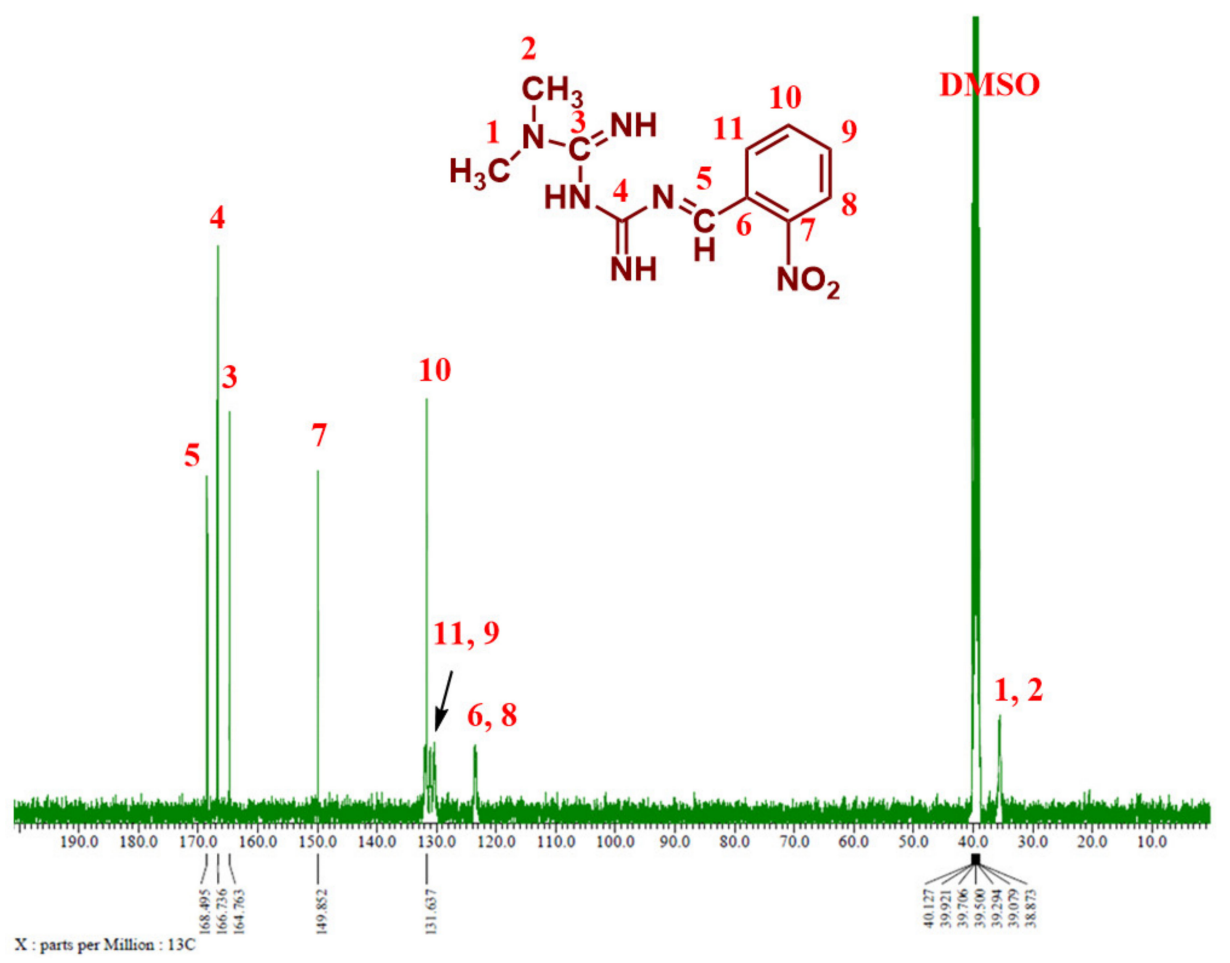
^13^C NMR spectrum of Comp1 in DMSO-*d*_6_.

**Figure 7:**
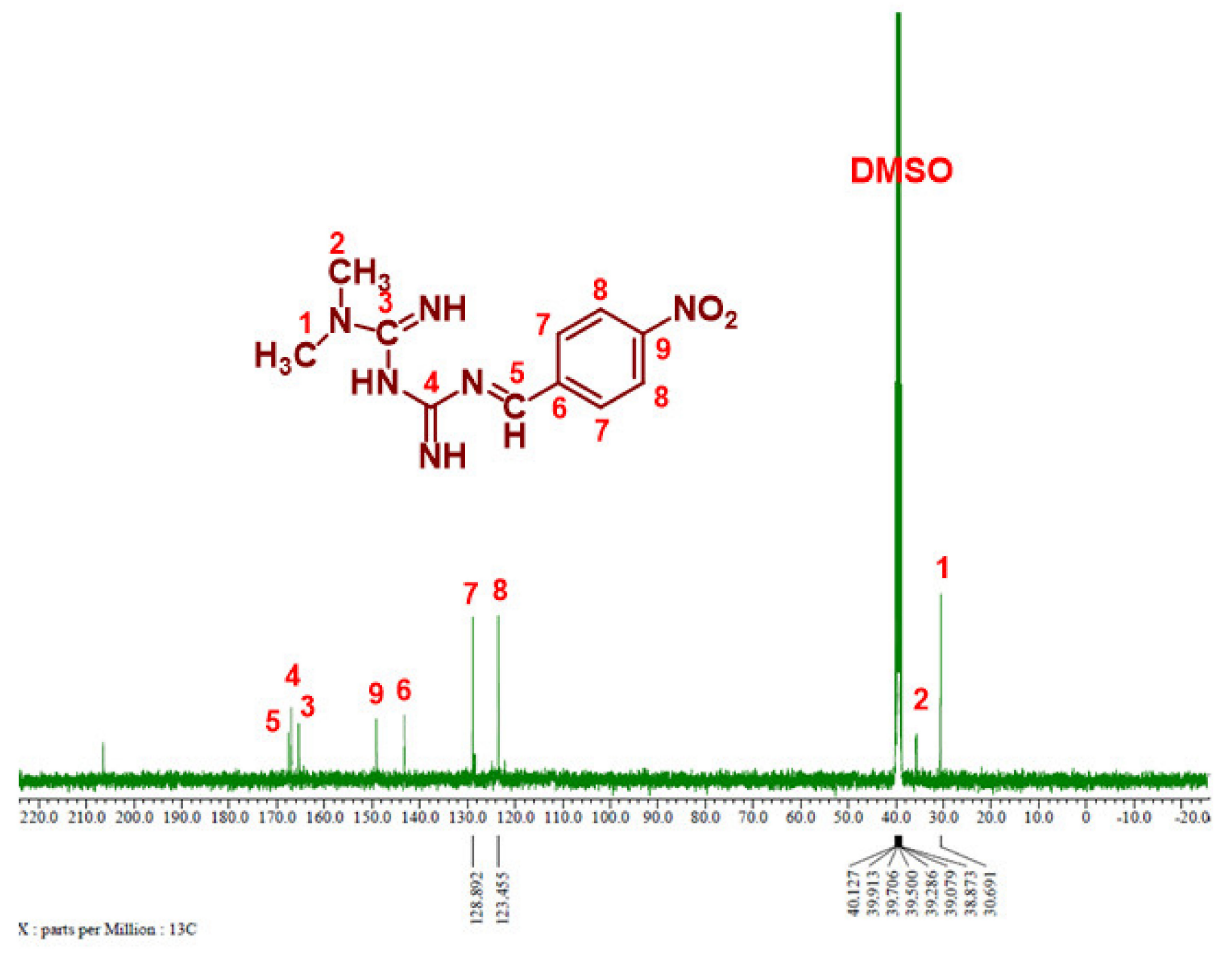
^13^C NMR spectrum of Compound 2 in DMSO-*d*_6_.

Figure 6 and Figure 7 show the ^13^C NMR spectra of Comp1 and 2, respectively. The chemical shifts corresponding to all compound carbons are identified, thus confirming their chemical structures. In both spectra, the characteristic peak of the aldehyde carbon that appears at 185–205 ppm disappears, confirming that the condensation reaction was successful. The peak corresponding to the new imine (C-5) is assigned at 168.46 ppm in both spectra. Despite their similar chemical formulas, the structural isomerization represented by the ortho- and para nitro substitution on Comp1 and 2, respectively, result in different chemical shifts for the same carbons, as shown in Figure 6 and Figure 7. In Comp2, C-7 and C-8 each represent two carbons existing in the same environment in the aromatic ring; thus, one peak was observed for each. The chemical shift corresponding to the nitro-attached carbons are observed at 149.85 (C-7) and 143.91 (C-9) in Comp1 and 2, respectively, reflecting the structural conformation shielding–deshielding effect. For easy comparison, the chemical shifts of all carbons are marked in the figures. The peak corresponding to DMSO (the NMR solvent) at about 39.55 ppm is dominant, resulting in a depression of some peaks such as C-6, C-8, C-9, and C-11 in Comp1, but identification is still possible [45,46].

#### 3.1.5. Mass Spectrum Analysis

**Figure 8:**
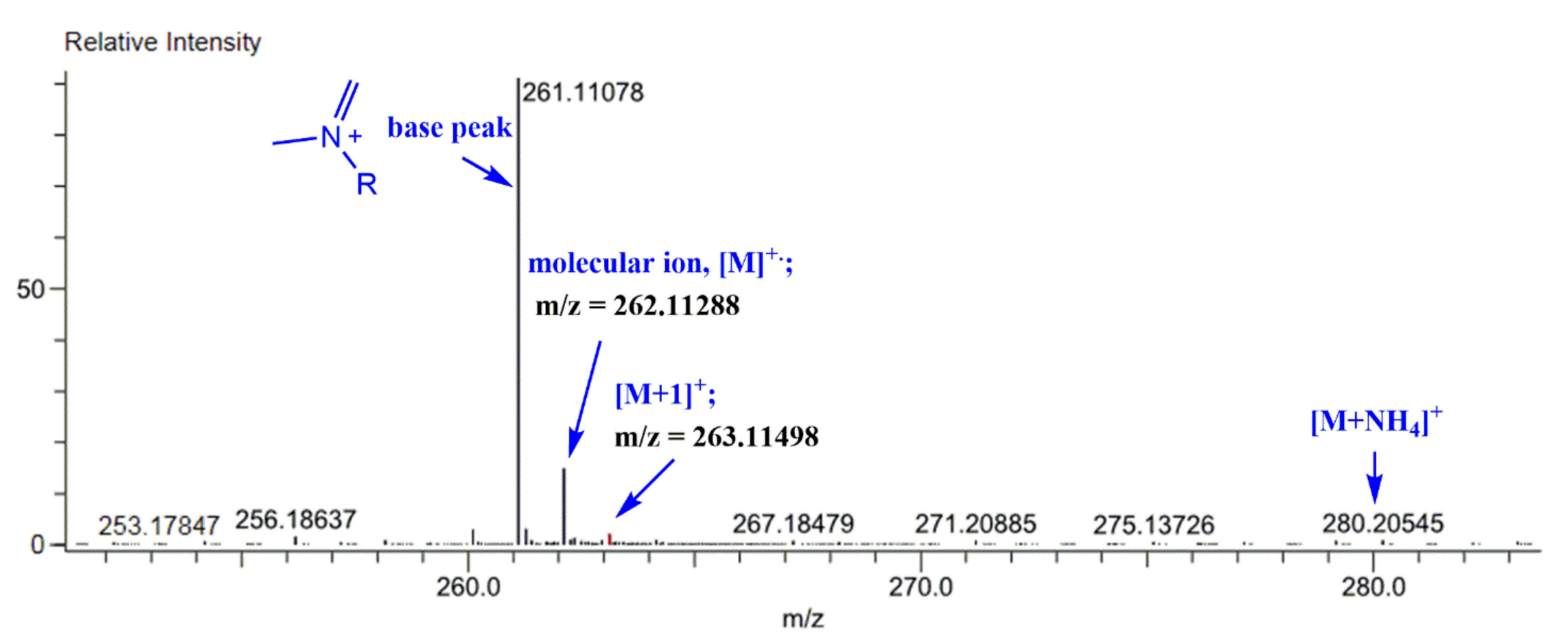
Mass spectrum of the synthesized Schiff base (Comp2).

**Scheme 2:**
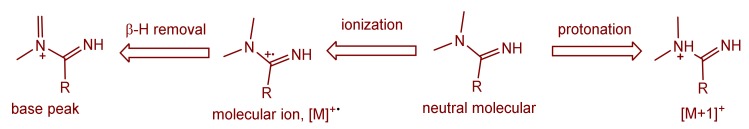
Suggested mass fragmentation route of the metformin-based Schiff base.

The mass spectra of the synthesized Schiff bases were also recorded. Figure 8 illustrates the mass spectra of Comp2 in which the peaks corresponding to the base peak, the molecular ion, the protonated molecule, and the ammonium adduct are observed at an m/z of 261.11, 262.11, 263.11, and 280.21, respectively. A similar fragmentation pattern for Comp1 is also seen. It is worth noting that *β*-hydrogen removal (a type of charge migration fragmentation (CMF)) is one possible route in a positive ionization mode that is induced by the presence of heteroatoms having a lone pair of electrons, resulting in the formation of a new π-bond that contributes to fragment stability possibly by resonance elongation, giving the most intense fragment peak (the base peak) (Figure 8) [47]. A suggested fragmentation pathway is illustrated in Scheme 2. The presence of the ammoniated adduct of the parent ion is another fragment proving the chemical structure of the compounds under investigation [48].

#### 3.1.6. Electronic Spectra

**Figure 9:**
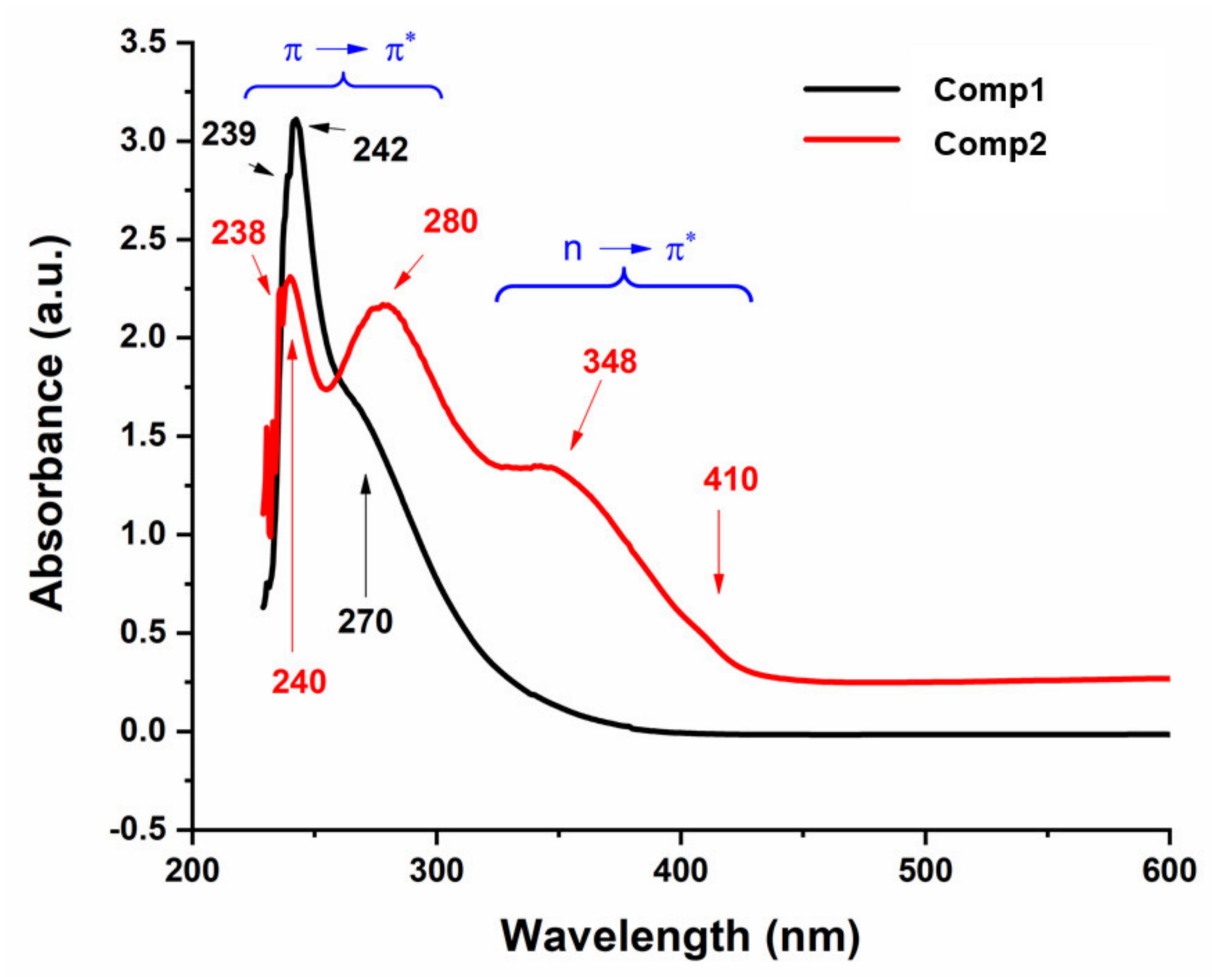
UV-Vis spectra of Comp1 and Comp2.

Figure 9 shows the UV-Vis spectra of Comp1 and Comp 2, which were recorded in 2:8 DMSO–water by volume at room temperature. Both compounds show electronic transitions a little below 450 nm (Figure 9). In the spectral range of 430–220 nm for Comp2, at least five bands are discernible with band intensity decreasing with increasing wavelength. This pattern is absent in the Comp1 spectra, in which only one distinct maximum is observed, which is associated with multi-unresolved shoulders. The solvent effect on the molecular absorption, as well as the high absorptivity value for Comp1, may rationalize such overlapping of the absorption peaks. According to the literature, the metformin electronic spectrum shows absorption maxima at 232 nm [49,50] that is attributed to the π–π* transition, while the nitro-substituted benzaldehyde spectra involve complexed multi peaks in the range of 200–400 nm [51]. The absorption corresponding to the n–π* transition of C=N and NO_2_ groups typically require less energy compared with the π–π* transition of the C=N and C=C groups existing in such materials (Figure 1). Therefore, the peaks below 250 nm could be attributed to the π–π*, while those above 250 nm could be attributed to the n–π* transitions. It can be observed that the n–π* excitation of *para*-isomers requires slightly higher energies compared with the *ortho*-isomer, while the opposite behavior is the case for the π–π* transition [51].

#### 3.1.7. Differential Scanning Calorimetry (DSC)

**Figure 10:**
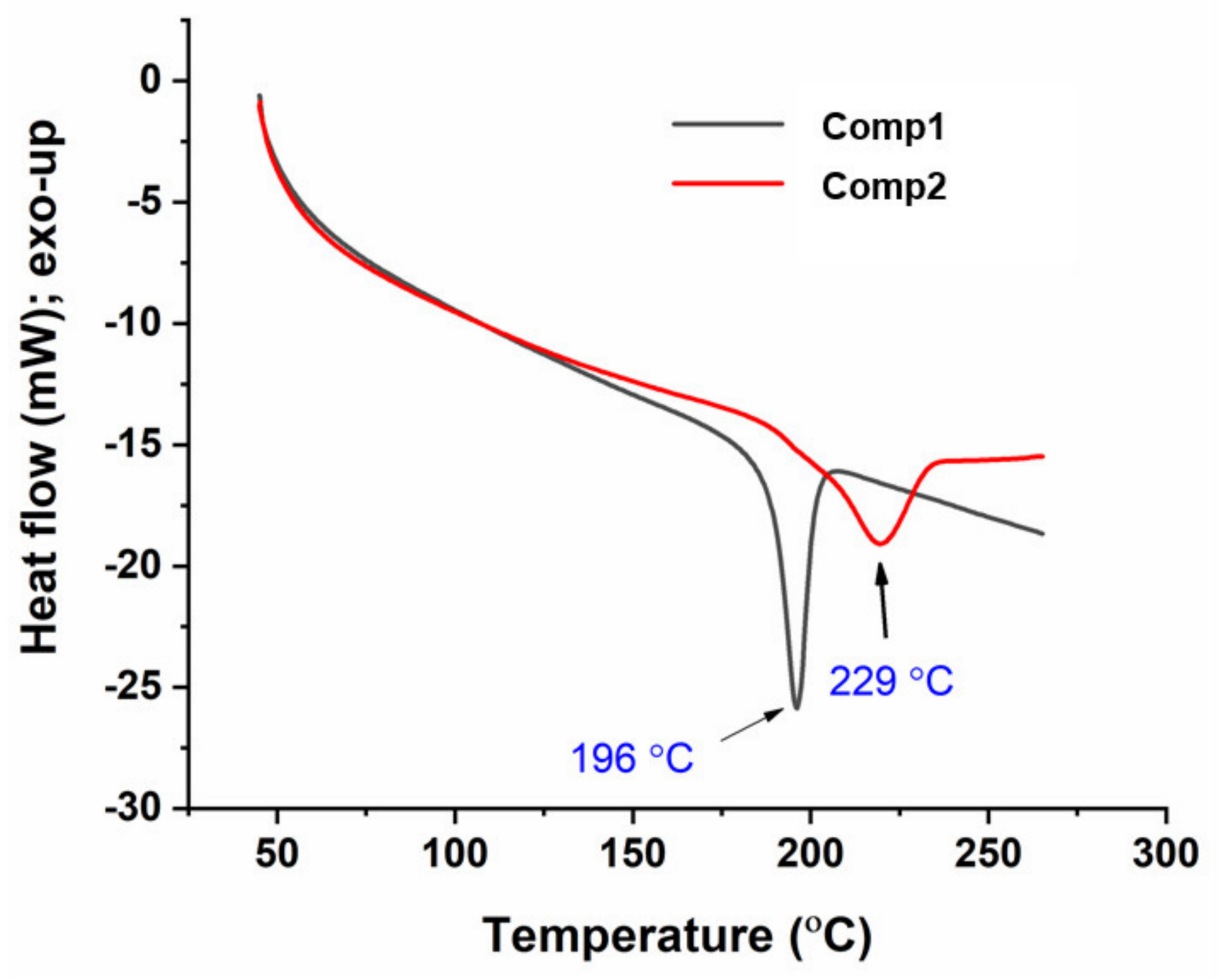
Differential scanning calorimetry (DSC) thermograms of Comp1 and Comp2.

Figure 10 shows the DSC thermograms of Comp1 and Comp2. In this figure, each compound exhibits a single, endothermic transition peak corresponding to its melting point at 196 °C and 229 °C, respectively, while the absence of additional peaks confirms the purity of the compound. The melting point measured by DSC and a digital melting-point apparatus (CL-726) is collected in Table 1. However, the values from DSC were 2–3 °C lower than those obtained by the CL-726. In such a case, the DSC-associated difference within the values of ± 2 °C can be tolerated, as the accuracy depends on various factors, including the heating rate and atmospheric conditions. The higher value for the Comp2 melting point is supposedly associated with its chemical structure. Hence, the planarity of the para-substituent may facilitate an intermolecular interaction compared with the non-planar ortho-isomer [51], resulting in a higher melting point for Comp2.

### 3.2. Biological Activity

**Figure 11:**
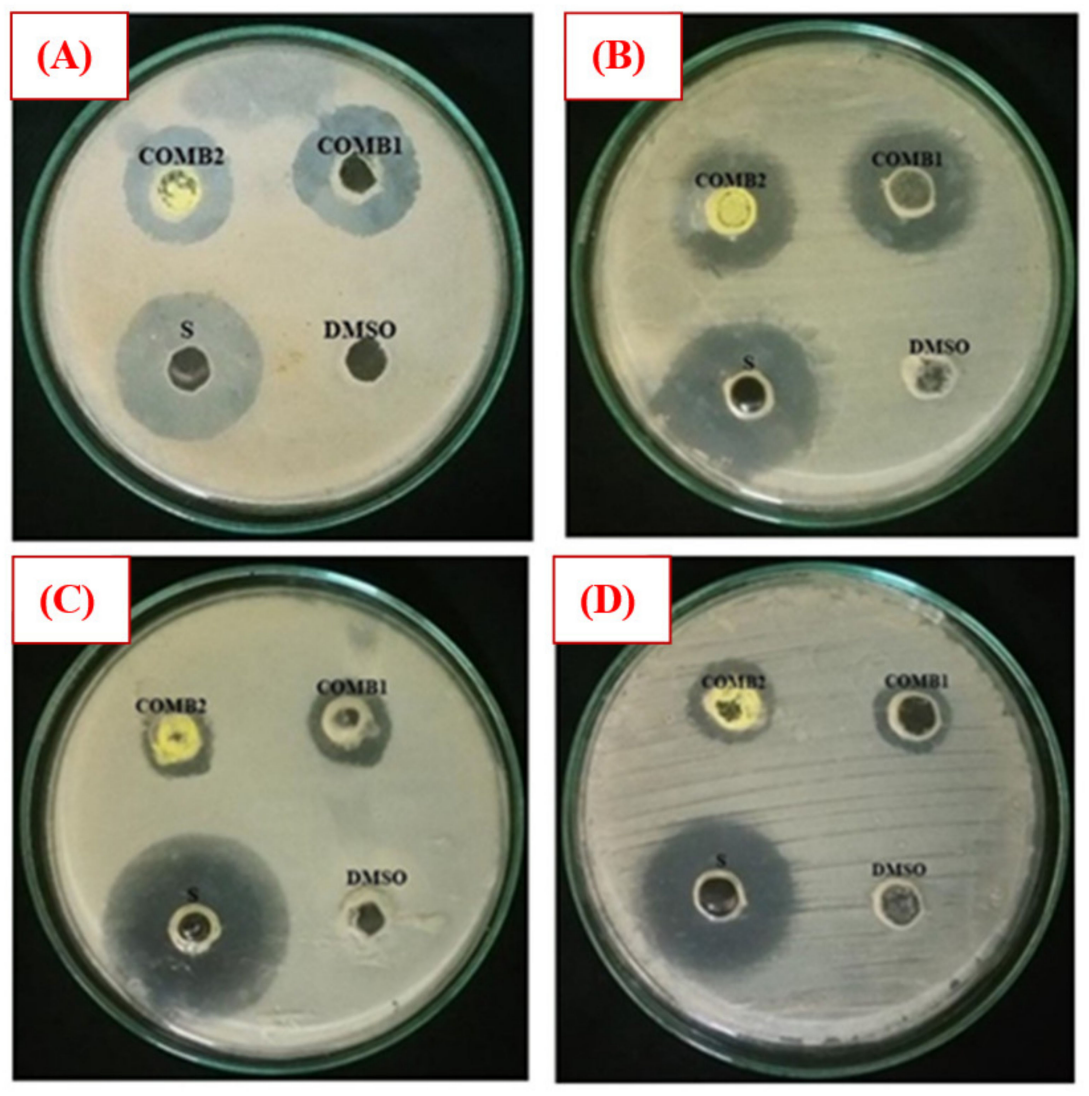
Selected plate images of antibacterial activity tests against (**A**) *S. aureus*; (**B**) *K. pneumonia*; (**C**) *E.* *coli*; and (**D**) *E.* *faecalis*. Comp1 = compound 1 (10 mg/mL); Comp2 = compound 2 (10 mg/mL); Dimethyl sulfoxide (DMSO) (negative control); S = standard (positive control) (1 mg/mL).

The antibacterial activity of Comp1 and Comp2 against some bacteria strains, including Gram-positive (*S. aureus*, *M-R S. aureus,* and *E. faecalis*) and Gram-negative (*E. coli*, *P. aeruginosa*, and *K. pneumoniae*) was investigated employing the agar well diffusion method. Experimentally, a range of concentrations of Comp1 and Comp2 was screened (1–10 mg⋅mL^−1^), and the lowest concentration showing an inhibitory effect was observed to be 10 mg⋅mL^−1^. The antibacterial activity was evaluated by measuring the observed inhibition zone diameter (Figure 11). Data from two independent experiments were averaged and reported in Table 2. According to the data obtained, Comp1 is more active against almost all of the tested bacterial strains compared with Comp2. However, the observed zone of inhibition (ZOI) for both compounds is smaller than the control (streptomycin), even though its concentration is 10 times lower. This result indicates that the standard antibiotic is more effective in its antibacterial activity than the tested compounds. In addition, Comp1 shows higher activity against *S. aureus* and *K. pneumoniae* with ZOIs of 24.33 and 23.67 mm compared with Comp2 with ZOIs of 20.67 and 20.67 mm, respectively, while a moderate inhibitory effect is observed for both compounds on *E. coli* and *E. faecalis* (Table 2). The results show that both compounds have no activity against *P. aeruginosa* and *MR S. aureus*. Images of selected experimental plates are presented in Figure 11.

## 4. Conclusions

Two new Schiff bases of metformin hydrochloride have been synthesized via newly established eco-friendly protocols using water as an eco-friendly solvent and a microwave-assisted process. The reaction exhibits good yields in short reaction times. The derivatives have been characterized by elemental analysis, FTIR, UV-Vis, ^1^H NMR, ^13^C NMR, and mass spectroscopy. Both compounds exhibited antibacterial activity against *S. aureus*, *K. pneumoniae, E. coli*, and *E. faecalis* with a noticeable higher activity of the ortho-isomer compared with the para-isomer, but less than the positive control (streptomycin). We conclude that the established methods are convenient and environmentally friendly, and the obtained metformin-nitrobenzaldehyde Schiff bases are effectively bactericidal; however, further work is needed to investigate their activity against other microorganisms as well as their efficacy as an insulin memetic and anticancer agents.

## Figures and Tables

**Table 1:** Data on preparation conditions and some analytical data for the metformin Schiff bases. The yields are reported as the isolated yields.

Compound	Color	Conventional Method		Stirring Method		Microwave Method		Melting Point (DSC) (°C)	Elemental Analysis Found (Calculated) (wt %)			
		Yield (%)	Time (min)	Yield (%)	Time (min)	Yield (%)	Time (min)		C	H	N	O
Comp1	Pale yellow	69	180	88	600	90	5	198 (196)	50.34 (50.38)	5.33 (5.38)	32.09 (32.04)	12.26 (12.20)
Comp2	Intense yellow	85	180	90	600	92	5	232 (229)	50.41 (50.38)	5.41 (5.38)	32.10 (32.04)	12.15 (12.20)

**Table 2:** Antibacterial activity inhibition zone of Comp1 and Comp2 on the tested bacterial species. All values are expressed as Mean ± SEoM (n = 2).

Bacterial Species		Diameter of Inhibition Zone (mm)		
		Comp1 (10 mg⋅mL^−1^)	Comp2 (10 mg⋅mL^−1^)	Streptomycin (1 mg⋅mL^−1^)
Gram-positive	*S. aureus* (ATCC 25923)	24.33 ± 0.33	20.67 ± 0.33	30.33 ± 0.33
	*MRSA* (ATCC 43300)	-	-	24.67 ± 0.33
	*E. faecalis* (ATCC 29212)	15.0 ± 0.58	17.67 ± 0.88	27.33 ± 0.33
Gram-negative	*E. coli* (ATCC 25922)	16.33 ± 0.33	14.67 ± 0.33	25.33 ± 0.33
	*K. pneumoniae* (ATCC700603)	23.67 ± 0.88	20.67 ± 0.33	32.67 ± 1.20
	*P. aeruginosa* (ATCC 27853)	-	-	20.33 ± 0.33
